# Metagenomic profiling of microbial communities and the resistome within Egyptian hospital wastewater and tap water

**DOI:** 10.1038/s41598-026-49481-4

**Published:** 2026-04-30

**Authors:** Hend Magdy Radwan, Nagwan Galal El Menofy, Engy K. Tharwat, Mohamed Mysara, Sahar Mohamed Ramzy Radwan

**Affiliations:** 1 Microbiology and Immunology Department. Faculty of Pharmacy (Girls), Al-Azhar University, Cairo, 11751 Egypt; 2 Bioinformatics Group, Center for Informatics Science, School of Information Technology and Computer Science, Nile University, Giza, 12588 Egypt; 3 Microbiology and Immunology department, Faculty of Pharmacy, Nile Valley University, Fayoum, 63518, Egypt

**Keywords:** Antibiotic resistant bacteria, Antibiotic resistance genes, Hospital wastewater, Microbial abundance, Nanopore sequencing, Resistome, Tap water, Biotechnology, Computational biology and bioinformatics, Microbiology

## Abstract

Antimicrobial resistance (AMR) is a worldwide health concern that compromises the successful treatment of a growing array of infectious diseases, particularly in low- and middle-income countries. AMR is exaggerated by the spread of antimicrobial resistance genes (ARGs) across humans, animals, and environmental reservoirs like water and soil. Hospital wastewater (HWW) is the main source of antimicrobial resistance in the environment. The current study used high throughput metagenomic nanopore sequencing to investigate the microbial abundance and ARGs associated with both HWW and tap water in five different hospitals in Cairo, Egypt. The bacterial community composition of the HWW microbiome identified 25 taxonomic families. The most abundant genera in HWW were *Acinetobacter* (6%) and *Propioniciclav* (5%) out of 101 unique genera while, the most abundant in tap water were *Enterococcus* (53%), *Escherichia* (15%), and *Francisella* (14%) out of 89 unique genera. Alpha diversity analysis revealed significantly greater microbial diversity in the HWW samples than in the tap water samples (*P* value > 0.05), moreover beta diversity analysis revealed a significant difference in the microbial community composition between the tap water and HWW samples (*P* value > 0.05) using Chao metric for richness estimation and Shannon metric for richness and evenness estimation. Total ARG analysis revealed absence of ARGs in tap water using the three databases, while comparable levels of ARGs were detected in HWW across the five hospitals. In total, 45, 28, and 28 ARG subtypes were identified in the HWW samples using ResFinder, CARD, and the NCBI AMRFinderPlus databases, respectively. The most abundant AMR mechanisms among the five hospitals were linked to the inhibition of protein synthesis. Using the ResFinder database, streptogramin resistance genes were most prevalent in Hospitals 1 and 5 (15% and 40%, respectively); using CARD, aminoglycoside, lincosamide, and macrolide resistance genes were most predominant (relative abundances 35–60%). Using NCBI AMRFinderPlus, streptomycin, tetracycline, and macrolide resistance genes were most prevalent (relative abundances 30.1–60%). Detection of plasmid replicons in HWW identified 39 different plasmid-associated replication genes via the PlasmidFinder database. The Col440l-1, colRNAI-1 and Col440ll-1 plasmid replicons were the most detected across the five hospitals with relative abundances of 16.6%, 10.9% and 9.6%, respectively. This study revealed different microbial communities among HWW and tap water in addition to the widespread occurrence of ARGs and AMR encoding plasmid replicons in the HWW in the five different hospitals in Cairo, Egypt indicating a significant risk associated with HWW, necessitating the implementation of preventative measures to avert their environmental diffusion. To our knowledge, this is one of the first Egyptian studies to apply Oxford Nanopore long-read metagenomic sequencing for simultaneous profiling of microbial communities and the resistome in HWW and tap water, using three ARG databases across five hospitals in two seasons.

**Supplementary Information:**

The online version contains supplementary material available at 10.1038/s41598-026-49481-4.

## Introduction

Antimicrobial resistance (AMR) is a global public health challenge that results in over 700,000 deaths annually. According to the World Health Organization (WHO), one of the main issues facing global health in the twenty-first century is antimicrobial resistance^1^. The widespread and increased use of antibiotics has resulted in the selection and enrichment of naturally occurring antibiotic resistant bacteria (ARB) and ARGs especially in the aquatic environment by antibiotics disseminated in sewage effluent and agricultural discharge^2,3^. Water is a significant mean of antibiotic resistance dissemination; multidrug-resistant (MDR) bacteria have been identified in a variety of water sources, even tap water and potable water^4^. Water interacts with everything and is connected to human-associated microbial communities^5^.

Historically, concerns about the microbial quality of drinking water have focused on the occurrence of pathogens in drinking water distribution systems. However, the presence of trace levels of antibiotics and ARB in source water and finished drinking water may also greatly affect public health since it increases the risk of mortality, decreases the effectiveness of treatment, and promotes the spread of resistant genes^6,7^. Several studies have detected ARB in drinking water systems but most of them focused on cultivable bacteria and indicator organisms^8–10^**,** Little is known about the fate of ARGs in drinking water systems as emerging contaminants^11^. As most previous studies on ARGs in aquatic environments used qPCR for quantification that selected only a few representative ARGs for detection^12,13^. However, more comprehensive detection of ARG profiles can yield deeper insights into the evolution of ARGs in aquatic environments.

HWW is the water released from all medical and non-medical hospital operations containing human waste, disinfectants, medical debris from surgical room and diagnostic lab^7,14,15^. Certain ARBs can even withstand disinfectants and are found in post-chlorination wastewater^16^. The prolonged and intensive use of antibiotics in hospitalized patients^17^ exerts strong selective pressure, promoting the enrichment of ARB and facilitating the horizontal gene transfer (HGT) of ARGs via mobile genetic elements including integrons, transposons, and plasmids^18,19^. Hospital effluent contains a variety of pathogenic bacteria, including *Escherichia coli, Pseudomonas aeruginosa,* and *Enterococcus* spp., which originated from contaminated medical equipment, healthcare personnel, or patient wastes^20,21^. The high rate of infection and toxicity of these bacteria make them extremely hazardous^22^.

HWW is a crucial hotspot for ARG dissemination, as reported by several studies. A study conducted in South America isolated 42 bacterial isolates from HWW, identifying multidrug-resistant strains harboring 56 ARG families and 38 virulence-factor families^23^. Similarly, a Japanese study demonstrated that hospital sewage was a significant reservoir of ARB and diverse ARGs^24^. Supporting these findings, a comprehensive systematic review encompassing 21 studies across Europe, Asia, Africa, and America confirmed that HWW consistently harbors a greater diversity and higher abundance of ARGs compared to other wastewater systems^25^, collectively establishing the hospital environment as a critical hotspot for ARG dissemination. Without appropriate treatment, ARBs or ARGs from clinical healthcare settings may spread and even flourish in the environment, hastening the emergence of MDR microorganisms^26^. Additionally, climatic conditions play a vital role in the growth and proliferation of microorganisms that prevail in waterbodies^27^. HWW treatment is important to stop the spread of ARB from the hospital to the surrounding environments^28,29^.

Antimicrobial susceptibility testing has emerged as the main method for evaluating bacterial resistance in recent years. However, phenotypic antibiotic susceptibility testing techniques have several limitations, such as reduced sensitivity, accuracy, and sample processing issues, which restrict our ability to study AMR. Molecular techniques are alternative methods based on the identification of known resistance genes. Although it can identify uncultivable bacteria and their ARGs, it is not appropriate for detecting novel ARGs as they rely on prior knowledge of the target gene sequence for primer or probe design and are therefore integrally limited to ARGs already catalogued in reference databases^30,31^.

Metagenomic sequencing is a useful technique that can be used for the wide-ranging detection of ARB and ARG to overcome the drawbacks of traditional and amplification-based approaches^32^. Simultaneous metagenomic studies, such as high-throughput shotgun sequencing of ARGs and mobile genetic elements (MGEs), enable a comprehensive understanding of microbial origin and development of ARG in HWW^33^. Long-read DNA sequences can be produced quickly with Oxford Nanopore Technologies which can provide lengthy reads that span the majority of the bacterial genome’s repetitive regions in average length of 10 kb^34^. Several studies have effectively employed Oxford Nanopore Technology for comprehensive investigation of AMR profiles and microbial communities in various environmental and clinical matrices^35–37^. The error rate of Nanopore sequencing can be overcome by several developed bioinformatic strategies that handle the underlying computational problems of error correction and de novo assembly of nanopore long reads^38^. Within the One Health framework, which recognizes the inextricable interconnection between human, animal, and environmental health, the dissemination of ARGs from hospital wastewater into aquatic environments represents a critical pathway through which clinically relevant resistance determinants can spread beyond healthcare settings, threatening ecosystem integrity and global public health^39^.

This study aimed to characterize the microbial community composition and diversity (α- and β-diversity) in HWW and tap water from five hospitals across Cairo, Egypt in summer and winter seasons, using whole-genome shotgun Nanopore sequencing. The parallel application of three ARG databases (ResFinder, CARD, and NCBI AMRFinderPlus) represents a key methodological contribution, enabling more sensitive and comprehensive resistome profiling than single-database approaches. This multi-hospital, multi-season design addresses a critical gap in the literature on hospital-associated AMR in Egyptian water systems, with direct implications for public health and wastewater management.

## Materials and methods

### Samples collection and processing

A total of 20 samples (10 tap water and 10 HWW) were collected from five hospitals distributed across different regions of Cairo, Egypt (Table 1), during two contrasting seasons: summer (August 2022) and winter (February 2023). Wastewater samples were collected directly from each hospital’s main drainage trench prior to reaching any municipal wastewater treatment facility; with no on-site wastewater treatment at the point of sampling. Tap water samples were sourced from the municipal water supply managed by the Cairo Drinking Water Company, which applies standard gaseous chlorination as the treatment process, maintaining residual chlorine levels within the government-permitted range of 0.2–0.5 mg/L. Regarding physicochemical conditions including pH, temperature, residual chlorine, turbidity, conductivity, and total organic carbon were not measured, which is added as a limitation of the present study.

**Table 1:** Distribution of hospitals under study.

Hospital	Location	Number of beds	Type
Hospital 1	Middle Cario	1200	Teaching and tertiary hospital
Hospital 2	North Cario	3430	Teaching and tertiary hospital
Hospital 3	South Cario	550	Teaching and tertiary hospital
Hospital 4	Middle Cario	1505	Teaching and tertiary hospital
Hospital 5	East Cario	350	General hospital

At each hospital, tap water (500 mL) was collected from a midstream flow point and HWW (100 mL) was collected from the public sink drain into sterile labeled bottles, following the grab sampling guidelines of the Environment Protection Authority^40^ and the American Public Health Association^41^. All the samples were placed in ice boxes and processed for analysis within 2 h. All water samples were filtered via 0.45 µm polyethylene sulfonate membrane filters (Sartorius Stedim Biotech, Sweden) using a closed laboratory filtration unit and stored in sterilized centrifuge tubes at − 80 °C till use.

### DNA extraction and quantification

DNA extraction from membrane filters was performed by DNeasy power water kit (Qiagen, Hilden, Germany), according to the according to the manufacture’s instruction for DNA isolation mentioned in the kit. DNA concentration and purity were assessed using a Qubit 4.0 fluorometer (Thermo Fisher Scientific, Waltham, USA). Samples with a minimum DNA concentration of 55 ng/µL, as required by the manufacturer’s protocol for the Oxford Nanopore Rapid Sequencing kit (SQK-RBK110.96), were considered adequate for downstream library preparation^42^..

### Library preparation and sequencing

Oxford nanopore (single-end library) sequencing was performed at HITS Solutions Co., Cairo, Egypt. Metagenomic sequencing library was generated via Rapid Sequencing gDNA-Barcoding (SQK-RBK110.96) (Oxford, England) according to the manufacturer’s protocol**.** Nine microliters of 55 ng template DNAand 1 µL rapid barcodes (one for each sample) were added. The barcoded samples were pooled, and amplicon purification was performed with AMPure XP Reagent. The beads were then washed with ethanol, and the residual DNA was eluted by adding 15 µl of elution buffer. Then, 11 µL of the sample was transferred and mixed with 1 µl of Rapid Adapter F (RAP F), which was then used for loading. The library was prepared by mixing 37.5 µL of sequencing buffer II and 25.5 µL of loading beads II with 12 µL of the DNA library. Priming and loading were carried out, and the library was subsequently loaded on an R.9.4.1 MinION flow cell for 12 h.

### Bioinformatic data analysis

MinKNOW software ver. 23.11.5 was used for data acquisition. MinION^TM^ sequence reads (i.e., POD5 data) were converted into fastq files via Dorado base call server ver. 7.3.9 (Oxford Nanopore Technologies) on AWS EC2 g4dn.xlarge. FASTQ data were first cleaned to remove any contaminating nonbacterial DNA using Kraken2, v, ref, which identifies the taxa associated with each sample via the mapping-based approach. The assembly-based approach was performed using the Flye assembler (v.2.9.4)^43^ utilizing the *– nanor-raw* parameter to reconstruct contigs and identify ARGs. ARGs inside the assembled contigs were identified by mapping the reads to the Resfinder database^44^, NCBI AMRFinderPlus database^45^, and CARD database^46^ by the ABRicate command-line program (v1.0.1) Galaxy Europe platform^47,48^. Moreover, the identified antibiotics were categorized according to their mechanism of action, and the taxonomic origin of each ARG was determined by mapping the assembled contigs with the NCBI nucleotide database via the BLASTn web server^49^. The relationships between ARG-related antibiotics and their respective taxa were illustrated via a chord plot generated via the GOplot R program^50^. The plasmid replicons within assembled contigs were also determined by mapping to the PlasmidFinder database^49^ within the ABRicate software on Galaxy Europe platform^46,48^.

### Statistical analysis

The operational taxonomic unit (OTU) table was rarefied to a depth of 30,000 and the data was imported to R. The alpha diversity indices were computed via the Chao^51^ and Shannon^52^ metrics for each sample via the Vegan R package (*alpha_meas*function, v.2.6)^53^, which reflects species richness and community complexity, respectively. Alpha diversity was followed by the Wilcoxon Rank-Sum test to validate the results statistically via* the Wilcoxon test* function in R (v.4.4.0). Beta-diversity was assessed using the Aitchison distance, computed on centered log-ratio (CLR)–transformed abundance data, and additionally using Bray–Curtis dissimilarity and the J-class index. Ordination and visualization of between-sample compositional differences were performed with the Reliability Centered Maintenance (RCM) framework [v.1.2]^54^. Differences in community composition between sewage and tap water samples were tested using PERMANOVA on the CLR-based distance matrix.

To reduce type I error, we applied very stringent cutoff combining ANCOMBC [v.2.8.1^55^, with (prev_cutoff = 0.04) with structural zero = TRUE (either Species that is completely absent in one of the conditions or differential abundant species), and then the identified significant differential abundant taxa was then parsed to Linear discriminant analysis effect size (LefSe)^56^ using microbiome marker software^57^ using CSS (Cumulative Sum Scaling) normalization approach and a linear discriminant analysis (LDA) cutoff of 3.5 to account for the significant compositionality difference. Cladogram was visualized utilizing galaxy online platform^58^.

Statistical analysis of antibiotic consumption rates among hospitals was conducted via the Kruskal‒Wallis test, followed by Dunn’s test for pairwise comparisons (using the *kruskal.test* and *dunnTest* functions, respectively, using Fisheries Stock Assessment (FSA) package in R^59^. To verify the assumptions for the Kruskal‒Walli’s test, normality was assessed via the Shapiro‒Wilk test and variance was checked via Levene’s test (using *the R functions of the Shapiro‒Wilk test and Levene’s test,* respectively). All visualizations were performed in R (v.4.4.0) via the ggplot2^60^, microbiome marker, GOPlot^50^ and vegan packages^53^.

## Results

### Reads, Alpha and beta diversity analysis of the HWW and tap water samples

Metagenomic sequencing generated a total of 307,471 and 18,356 clean sequence reads from HWW and tap water samples in summer, and 307,813 and 22,780 reads in winter, respectively, indicating comparable sequencing depth across both seasons. Bacterial sequences dominated the metagenomic profiles in all samples across both seasons, accounting for more than 99% of total classified reads in HWW and 97.5% in tap water. Non-bacterial microorganisms, including viruses, fungi, and archaea, collectively represented less than 1% and 2.5% of total reads in HWW and tap water, respectively. A complete breakdown of classified read counts by microbial community for all samples in two seasons is provided in Supplementary Table S1.

**Fig. 1:**
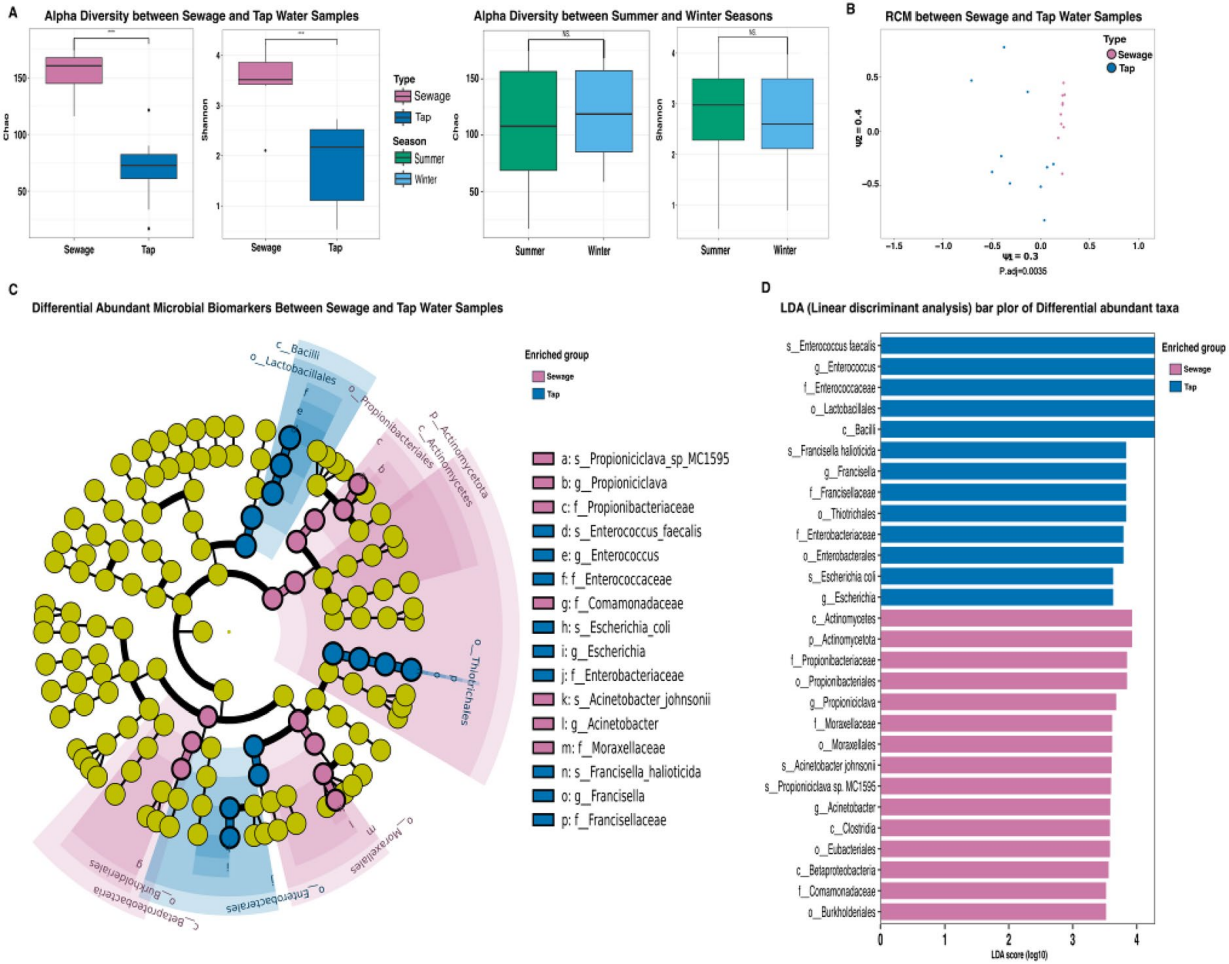
Diversity measurements across tap water and HWW (**A**) Box plots for evaluation of the alpha diversity via the Chao and Shannon indices, among HWW and tap water samples (Left Panel) and seasonal variation measurements (Right panel) (**B**). Principal component analysis (PcoA) plot utilizing the RCM method for assessing beta diversity between the HWW and tap water samples. (**C**). Linear discriminant analysis (LDA) bar plots represent differentially abundant taxa in related taxonomic ranks between the HWW and tap water samples. (**D**). Cladogram representing differentially abundant taxa and related taxonomic ranks between the HWW and tap water samples.

Alpha diversity analysis was performed via two indices, the Chao1 estimator and the Shannon index. The Chao1 estimator, which emphasizes species richness, demonstrated significant diversity in the HWW samples than in the tap water samples. Similarly, the Shannon index, which incorporates both richness and evenness, revealed significantly greater bacterial diversity in HWW than in tap water (*p-value* < 0.0001) (Fig. 1 (A). For assessing differences in microbial communities, beta diversity was used, utilizing the RCM compositionality-aware method, followed by PERMANOVA for statistical validation. The RCM plot revealed segregation between the tap water and HWW samples along the primary axes (ψ1 = 0.4 and ψ2 = 0.29), which revealed significant differences in the microbial community composition, as the points of these two groups were scattered (*p-value* < 0.005; Fig. 1B; Supplementary Fig. 1).

### Bacterial community characteristics in HWW and tap water

Following our stringent cutoff applied for differential abundance analysis, the over-represented genera in the HWW samples were *Acinetobacter* (relative abundance = 6%; P-adjusted = 0.03) and *Propioniciclav* (relative abundance = 5%; BH-adjusted p-value = 0.003). In contrast, the most representative genera in the tap water samples were *Enterococcus* (relative abundance = 53%; BH-adjusted p-value = 0.008), *Escherichia* (relative abundance = 15%; BH-adjusted p-value = 0.001), and *Francisella* (relative abundance = 14%; P-adjusted = 0.002) (Fig. 1 (C & D; Supplementary Table S1, Supplementary Table S2). For analysis on seasonal variation level**,** Alpha diversity measurements by the Chao and Shannon indices revealed nonsignificant differences in the abundances of microbial communities among the HWW samples in the summer and winter seasons (Fig. 1A).

### Detection of antibiotic resistance genes in HWW using different databases

The assembled contigs were mapped to the ResFinder, CARD, and NCBI AMRFinderPlus database to identify ARGs within each sample. In tap water, no ARGs were detected in the five hospitals across the three databases. For HWW, ResFinder database detected 1364 subtypes of ARGs including 553, 121, 120, 160, and 410 ARGs in the wastewater of hospitals no.1, 2, 3, 4, and 5, respectively.

**Fig. 2:**
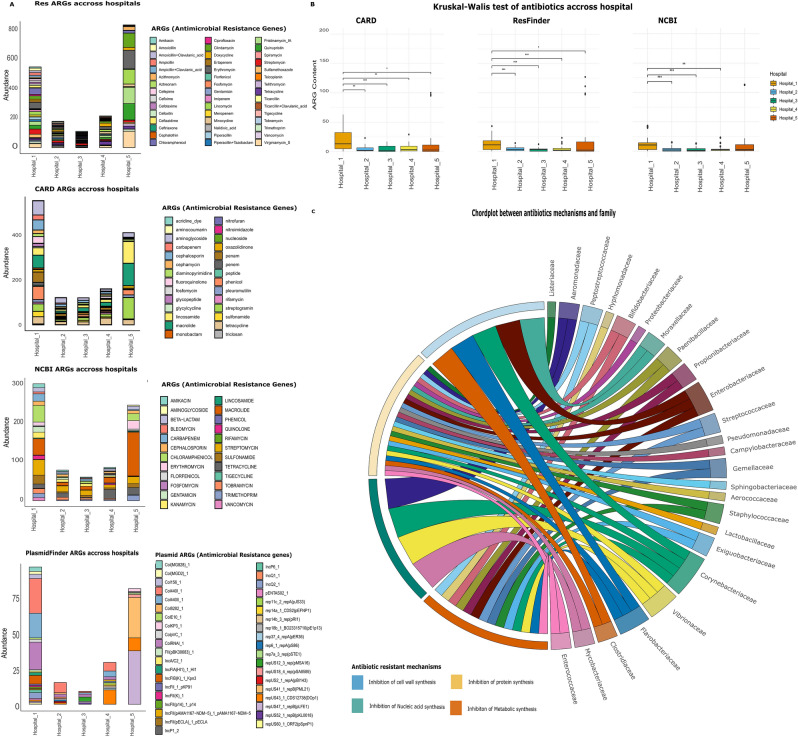
Relationship between the microbiome and the resistome among the five hospitals (**A**): (from top to the bottom): ResFinder stacked bar plots analysis of ARGs in HWW, CARD bar plots analysis of ARGs in HWW, NCBI AMRFinderPlus bar plots analysis of ARGs in HWW, Plasmid detection of HWW samples via the PlasmidFinder database (**B**): Box plot of the Kruskal‒Wallis significance test for ARG content detected using ResFinder, CARD, and NCBI AMRFinderPlus databases in HWW samples across the five hospitals. (**C**): Chord plot represents the relationships between each bacterial family and their associated mechanisms of action.

Using the ResFinder database, a total of 45 antibiotic resistance gene (ARG) types were identified in the hospital wastewater (HWW) of the five study hospitals, as illustrated in Fig. 2A. In Hospital 1, streptogramin resistance genes were the most prevalent, accounting for 15% relative abundance and represented by virginiamycin-S, quinpristin, and pristinamycin-1A, followed by erythromycin and chloramphenicol resistance genes, each comprising 8% relative abundance (Supplementary Table S3). Hospital 4 exhibited a notably higher abundance of ARGs associated with tetracycline and doxycycline resistance, both at 21.3% relative abundance. In contrast, Hospital 5 was dominated by streptogramin resistance genes (40% relative abundance), followed by erythromycin resistance genes (15% relative abundance), which collectively represented the most abundant ARG classes detected at that site.

Using the CARD database, 28 ARG types were identified in the HWW across the five study hospitals, as shown in Fig. 2A. Resistance genes associated with aminoglycosides, lincosamide, and macrolides were the most predominant ARG classes detected across all five hospitals, with relative abundances ranging from 35 to 60%. Furthermore, ARGs conferring resistance to phenicols, carbapenems, cephalosporins, streptogramins, and penems were notably abundant in Hospital 1, with a collective relative abundance of 37.1% (Supplementary Table S3). In Hospital 5, streptogramin, lincosamide, and macrolide resistance genes were the dominant ARG classes, reaching a relative abundance of 71.2%.

Using the NCBI AMRFinderPlus database, 28 ARG types were identified in the HWW across the five study hospitals, as shown in Fig. 2A. Resistance genes associated with streptomycin, tetracycline, and macrolides were the most predominant ARG classes detected across all five hospitals, with relative abundances ranging from 30.1% to 60%. In Hospital 1, ARGs conferring resistance to chloramphenicol, macrolides, sulfonamides, and streptomycin were the most abundant, reaching a relative abundance of 49%. In contrast, macrolide and erythromycin resistance genes were the dominant ARG classes in Hospital 5, with a relative abundance of 55% (Supplementary Table S3).

All three databases detected a higher level of ARGs within hospital 1 followed by hospital 5 samples. Compared with all other hospitals, hospital 1 exhibited a statistically significant difference in ARG content across the ResFinder, CARD, and NCBI AMRFinderPlus database according to the Kruskal‒Wallis test accompanied by Dunn’s post hoc test (Fig. 2B and Supplementary Table S4).

Our study revealed that the most abundant ARGs among the five hospitals compared with all three databases were linked to protein synthesis inhibitors, such as aminoglycosides *(AAC(6')-Ib9, ANT(3'')-IIa, aadS, APH(3'')-Ib, APH(6)-Id, ANT(4')-Ib, AAC(6')-Ie-APH(2'')-Ia, ANT(6)-Ia), lincosamides (ErmF, ErmB, mel, ErmG, ErmT, ErmA, ErmX*), tetracycline (*tetM, tetL, tetW*) and macrolides (*ErmB, mel, ErmG, ErmA, ErmX*) (Supplementary Table S3).

Regarding each hospital, ARGs conferring resistance to chloramphenicol (*rpoB, mel, cmx, and cmLA5*) and vancomycin (*vanR-A*) were most abundant in hospital 1 with 28.6% and 7.1% Relative abundance respectively out of 14 unique resistance-associated genes identified at this site. Moreover, Hospital 1 and hospital 5 contained ARGs associated with streptogramin (*ErmB, mel*, *ErmG, ErmA, ErmX, ermT*) collectively represented the highest relative abundance (42.9%), followed by fluoroquinolone resistance genes (*QnrVC4* and *qacH*; 14.3%), sulfonamide (*sul1*; 7.1%), and carbapenem (NDM-1; 7.1%) in Hospital 1. In Hospital 5, a total of 17 unique resistance-associated genes were detected, with streptogramin resistance genes (*ErmB, mel, ErmG, ErmA, ErmX, ermT*) again being the most prevalent (35.3%), followed by chloramphenicol (*rpoB, mel, cmx, cmLA5*; 23.5%), while trimethoprim (*dfrG, dfrA14*), cephalosporin (OXA-10, OXA-212), and fluoroquinolone (*QnrVC4, qacH*) resistance genes each accounted for 11.8% relative abundance. Sulfonamide (*sul1*) and carbapenem (NDM-1) resistance genes were present at 5.9% each in Hospital 5.

### Seasonal variation in ARGs in the HWW samples

There was an increase in the ARG diversity in the HWW samples among the five hospitals in summer compared with that in winter according to the ResFinder database. Hospital 5 presented high levels of ARGs against carbapenem (imipenem, meropenem, and ertapenem), β-lactam antibiotics (amoxicillin, ampicillin, piperacillin, ceftazidime, cefotaxime, and aztreonam) and ciprofloxacin in summer compared with those in winter. Hospital 1 exhibited high ARGs against the antibiotics azithromycin, doxycycline, and trimethoprim in summer compared with those in winter. Compared with those in winter, the diversity of ARGs in hospital 3 was greater in summer, and the levels of ARGs against streptomycin, erythromycin, and β-lactam antibiotics in summer were greater. The ARGs against streptogramin (virginiamycin-S, quinpristin, and pristinamycin-1A), lincomycin, erythromycin, and clindamycin antibiotics were most abundant in winter among hospital 4 and hospital 5, whereas the ARGs against streptomycin were most abundant in summer among the five hospitals (Supplementary Fig. 2).

### Detection of plasmid replicons in HWW samples via the PlasmidFinder database

The PlasmidFinder database was used to detect the plasmid replicons in the sequenced reads of HWW samples across the five hospitals. A total of 39 different plasmid-associated replication genes were detected. The most detected plasmid replicons were Col440l-1, colRNAI-1 and Col440ll-1with relative abundances 16.6%, 10.9% and 9.6%, respectively (Fig. 2A). Moreover, several plasmid replicons were detected. Greater abundances of col440l-1 (25.2%), col440ll-1 (17.8%), colRNAI-1 (20%), IncFIB (k)−1-Kpn3 (6.3%), and Incq-1 (4.2%) plasmids were detected in hospital 1, while in hospitals 2 and 3, col440l-1 (46.6%)—colRNAI-1 (13.3%) plasmids, and repUS12-3-rep (PMSA16)−1 (33.3%)—colRNAI-1 (22.2%) plasmids, respectively, were highly abundant. Moreover, hospitals 4 and 5 presented greater abundances of repUS43-1-CDS12738(Dop1) (34.4%)—col440l-1 (20.6%)—col440ll-1(13.7%)—colRNAL-1 (6.8%) plasmids, and repUS47-1-repB(PLFE1) (46.2%)—repUS41-1-repB (PML21) (35%)—repUS43-1-CDS12738(DOp1) (11.2%) plasmids, respectively (Supplementary Table S5).

## Linkage between each bacterial family and their associated mechanisms of action

After the categorization of the identified ARG products based on their mechanism of action, four main mechanisms were associated with 25 related taxonomic bacterial families. The mechanisms were ordered from the most to the least as follows: inhibition of protein synthesis, inhibition of metabolic pathways, inhibition of nucleic acid synthesis, and inhibition of cell wall synthesis. Members of the *Corynebacteriaceae* family were involved in all four mechanisms of action, illustrating their extensive functional role. Conversely, members of the *Enterobacteriaceae* and *Flavobacteriaceae* families participate in all mechanisms except for the inhibition of nucleic acid synthesis. Other families and their respective members exhibited relationships with only one or two particular mechanisms, as illustrated in Fig. 2C; Supplementary Table S6.

## Discussion

The emergence and spread of ARB, and ARGs in the environment expose patients and populations to serious risks^61^. HWW acts as a substantial reservoir and pathway for AMR by introducing sub-lethal antibiotic metabolites into the water system, hence facilitating the environmental entry of antibiotics with subsequent emergence of AMR. In the current study, we investigated the diversity and the abundance of bacterial communities and ARGs in HWW and tap water in five main Egyptian hospitals via whole genome nanopore sequencing analysis.

In this study, alpha diversity analysis via the Shannon and Chao indices revealed significantly greater diversity in HWW than in tap water. Additionally, beta diversity showed a significant difference in the microbial community composition between the two groups. This low microbial diversity in tap water can be explained by effective chlorine decontamination of tap water in Egypt. On the other hand, HWW contains hospital wastes, such as the excrement of patients, chemicals, pharmaceutical residues, and human pathogens, which create high microbial diversity and bioburden^62^. The community abundance of bacteria in the HWW among the five hospitals revealed the overrepresentation of genera, *Acinetobacter* (6%) and *Propioniciclav* (5%), in addition to the families propionibacteriaceae and Moraxellaceae. These genera mainly emerged from the human digestive tract^63^. These findings were similar to a study by Guo et al*.,* (2017) in China, where *Arcobacter*, *Acinetobacter*, *Bacteroides*, *Prevotella*, and *Bifidobacterium* were the most abundant genera in HWW^33^. Similarly, another study by Wang et al*.,* (*2*018) revealed that the predominant phyla were *Arcobacter*, *Acinetobacter*, *Dechloromonas*, and *Prevotella*, which were identified with different relative abundances^26^. A Brazilian study performed by Perry et al*., (2*021), reported that environmental species like *Pseudomonas fluorescens* and *Acinetobacter johnsonii* were the most common genera in HWW^65^***.*** In contrast, a study by Cai et al*.,* 2021 revealed that the main bacterial community compositions of hospital sewage using metagenomic techniques were *Escherichia coli*, *Hydrogenophaga pseudoflava*, *Reyrenalla massiliensis, K. pneumoniae, Acidovorax* sp. KkS102, *Protus mirabilis* and *Pseudomonas aeruginosa*^66^.

The genus *Propioniciclav* is classified as a part of the Propionibacteriaceae family which, constitutes a phylogenetically coherent family isolated from different habitats, including activated sludge, the marine environment, contaminated soil, and human samples^67^. Furthermore, the family Moraxellaceae comprises species that invade mucosal membranes and can occasionally cause a range of infections, as well as harmless species that inhabit the environment, including water, soil, and food^68^.. *S*everal *Acinetobacter* species are found naturally in different environments, including soil, water, air, wastewater, fomites, human skin, animals, and even plants^69^*. Acinetobacter johnsonii* is a common inhabitant of the intestinal tracts of many animals and rarely causes human infections. The relatively high abundance of *A. johnsonii* bacteria in HWW was mainly due to its ability to form biofilm in hospital pipes^65,70^.

The nonsignificant differences in the abundances of microbial communities among the HWW samples in the summer and winter may be explained by environmental buffering within enclosed hospital drainage systems, which maintains more stable temperature and humidity conditions compared to open water bodies, in addition to the continuous and relatively constant input of human-derived organic matter, carbon, and nitrogen sources from hospital activities throughout the year, which sustains stable microbial community structure regardless of season^71,72^.

The bacterial community abundance in the hospital tap water from the five hospitals revealed that.

most representative genera in the tap water samples were *Enterococcus* (53%), *Escherichia* (15%), and *Francisella* (14%). Members of the Enterobacteriaceae family, including *Escherichia coli*, are recognized as possible pathogens present in soil and water. The presence of enteric bacteria, such as *Escherichia coli* and *Enterococcus faecalis*, serves as a reliable indicator of fecal contamination in water sources. This contamination poses significant public health risks, primarily due to the potential for severe diarrheal infections and the spread of antibiotic-resistant pathogens. Furthermore, when production animals consume water from these contaminated sources, it can facilitate the widespread transmission of microorganisms within livestock populations^73^.

The genus *Francisella* is remarkably diverse, with numerous species naturally occurring in soil, water, and on plants^74^. In 2005, *Francisella halioticida* was identified as a notable pathogen affecting mollusks^75^. The current study represents the first documented instance of a *Francisella* species being detected in tap water; consequently, further research is strongly encouraged to investigate these environmental isolates and better understand their diversity and transmission patterns*.* A study conducted by Mulamattathil et al*.,* (2014) identified environmental bacteria from various raw drinking water sources in South Africa, where fecal and total coliforms were detected in the treated water samples^76^. Similar to our finding, a study carried out by Deji-Agboola et al*.,* (2014) in Nigeria identified coliforms in tap water samples, including *Escherichia coli, Klebsiella* spp*., and Enterobacter* spp*.*^77^. In contrast, Brumfield et al*.,* (2020) carried out shotgun metagenomic analysis to determine the microbiological content of drinking water and reported that the members of Actinobacteria and Proteobacteria, were the predominant bacterial species whereas fecal indicator bacteria such as *Escherichia coli* or *Enterococci* were not detected^78^.

In the current study, no ARGs were detected in tap water in the five hospitals by the three used databases, which indicates efficient chlorination treatment of tap water in Egypt which eliminates ARB and degrades extracellular DNA containing ARGs. In addition, the tap water was collected from storage tanks and water storage frequently leads to biomass decay, which breaks down extracellular DNA that may contain ARGs^79^.

In HWW, a relatively high level of ARGs within all hospitals was detected. Hospital 1 exhibited a statistically significant difference in ARGs compared with all other hospitals across the three databases. The ResFinder database detected 45 types of ARGs in the HWW, whereas the CARD and NCBI AMRFinderPlus databases detected 28 types. ResFinder database is a well-curated database of mobile and acquired ARGs which may explain the greater detection rate of ARGs^44,80^. Additionally, it contains highly accurate resistance phenotypes for well-studied organisms, and the ARGs per antimicrobial class in FASTA format^81^.

Furthermore, our analysis demonstrated that the most abundant ARGs among the five hospitals were associated with antibiotics that inhibit protein synthesis, such as aminoglycosides, tetracycline, and macrolides. Our findings are consistent with findings by Talat et al*.,* (2023) in India, which revealed that many of the identified ARGs in HWW samples were aminoglycoside-modifying enzymes, carbapenemase, trimethoprim resistance gene *dfrA1*, macrolide-lincosamide-streptogramin (MLS) resistance gene; and sulfonamide resistance gene^82^. Similarly, a study carried out by Wang et al*.,* (2018) using high-capacity qPCR revealed that the most identified ARGs were associated with tetracycline-resistance genes (*tetX, tetM, tetO*); macrolide resistance genes (*ereA, ermA, ermB*); sulfonamide-resistance genes (*sul1, sul2, sul3*) and quinolone-resistance genes (*qnrA, qnrB, oqxB*)^64^. Also, similar to our study, Cai et al*.,* (2021) found that ARGs for aminoglycosides were the most common, followed by sulfonamide, tetracycline, phenicol, macrolides and quinolones, comprising 82.6% of all ARGs^66^. In contrast, a study carried out by Guo et al*.,* (2017) revealed that the most abundant ARGs, were associated with bacitracin, tetracycline, and tobramycin^33^.

The observed inter-hospital differences in microbial community composition and ARGs abundance are likely attributable to several factors: (i) differences in patient population and disease burden among hospitals (e.g., Hospital 1 is a large tertiary teaching hospital with 1,200 beds, while Hospital 5 is a smaller general hospital with 350 beds); (ii) differences in antibiotic usage patterns and prescribing practices between hospitals; and (iii) the geographic distribution of hospitals across different regions of Cairo, which may influence the background environmental microbiome.

The WHO has classified the dissemination of ARGs and their prevalence on mobile genetic elements as an urgent health concern due to their direct association with the failure of hospital antibiotic treatments^83^. A possible explanation for the observed increases in ARGs among the five hospitals may be related to the high use of these antibiotics in the hospitals and their subsequent release into the environment. It was found that many clinical ARGs were reported to emerge from non-pathogenic hosts through HGT^84,85^. Also, it was found that pathogens in hospital sewage were able to rapidly acquire additional and fully functional ARGs through HGT^66^. Regarding the seasonal difference in both ARG abundance and composition in our study, our detection was similar to Honda et al*.,* (2023)^86^. However, different studies have not confirmed the obvious seasonal fluctuations in the occurrence of ARGs detected in wastewater treatment systems^87^. These seasonal variations and greater diversity of ARGs in summer can be explained by factors such as higher water temperatures, longer periods of sunshine, increased nutrient availability, and more frequent human activities, which may contribute to bacterial pathogen proliferation and dissemination^88^.

In our study, the PlasmidFinder database was used to predict the plasmid replicons in the sequenced reads. The most prevalent plasmid replicon type belonged to the Col family. Three types of Col plasmid replicons were observed, namely, ColRNAI, Col440II, and Col440I, which were detected in the five hospitals with relative abundances of 16.6%, 10.9% and 9.6%, respectively. Col plasmids contained genes that encode bacteriocins and proteins that can kill other bacteria^89^. Attalla et al*.,* (2023) detected three types of Col plasmid.

replicons (ColRNAI, Col440II, and Col440I) that were present in *Klebsiella* spp. obtained from hospital laboratory^89^. Furthermore, there was a greater abundance of repUS43-1-CDS12738 (DOp1) plasmid in hospitals 4 and 5. In addition, hospital 5 presented increased abundances of the repUS41-1-repB (PML21) and repUS47-1-repB (PLFE1) plasmids. Analysis of the complete closed genomes revealed that repUS43 is chromosomally integrated and is proximal to a tetracycline resistance gene (*tetM*). A study conducted by Sharon et al*.,* (2023) revealed that the most common plasmid replicon type was repUS43, which was found in *Enterococcus faecalis* in patients with urinary tract infection^90^.

The detection of 39 distinct plasmid replicons in HWW is of considerable clinical and epidemiological significance. Plasmids are key vectors of HGT, enabling the rapid spread of ARGs between bacterial species and across environmental compartments^91^. The dominance of Col-type plasmid replicons (Col440I-1: 16.6%, ColRNAI-1: 10.9%, Col440II-1: 9.6%) is particularly notable, as these small mobilizable plasmids are frequently co-transferred with larger resistance plasmids and can facilitate the dissemination of ARGs across Gram-negative pathogens. The presence of IncFIB(K) and IncQ plasmids in Hospital 1 is especially concerning, as these incompatibility groups are commonly associated with carbapenemase-encoding plasmids in *Klebsiella pneumoniae* and represent a significant threat of carbapenem resistance spread^92,93^. A study of *Klebsiella pneumoniae* species complex plasmids shows a highly diverse and ecologically adaptable plasmidome^92^. The co-detection of NDM-1 in Hospital 1 alongside these plasmid replicons further underscores the risk of ARG dissemination from HWW into the wider environment^91^.

Based on the correlation between each bacterial family and their associated mechanisms of action, four main mechanisms associated with the twenty-five related taxonomic families were identified. Members of the Corynebacteriaceae family are involved in all four mechanisms of action, whereas members of the Enterobacteriaceae and Flavobacteriaceae families participate in all mechanisms except for the inhibition of nucleic acid synthesis. The Corynebacteriaceae family contains more than 80 species isolated from diverse backgrounds, such as human clinical samples, animals, soil, marine environments, and dairy products. The outer membrane of these bacteria acts as an exterior permeability barrier, which explains the increased antibiotic resistance of these organisms^94^.

The current investigation revealed a significant difference in bacterial diversity and the alarming presence of elevated ARGs in HWW compared to their absence in tap water. There was a greater prevalence of ARGs for macrolide, aminoglycoside, tetracycline, and lincosamide antibiotics in the HWW samples across the five hospitals indicating a significant risk associated with HWW and requiring the implementation of preventative measures to prevent their environmental diffusion.

Although the results of our study indicate wide range of ARGs distributed among the five hospitals, it is likely that other factors like the physicochemical properties may affect the abundance of ARGs. Therefore, further research is needed to better understand these factors. In addittion, the *Escherichia coli* and *enterococcus* species detected in tap water should be isolated in order to characterize them genotypically.

Despite these findings, several limitations of the present study warrant consideration in future work. First, absence of physicochemical water quality parameters (temperature, pH, conductivity, turbidity, residual chlorine), which limits mechanistic interpretation of microbial community structure. Sconed, insufficient sequencing coverage in tap water samples, which may have reduced the sensitivity to detect low-abundance ARGs and precluded robust metagenome-assembled genome (MAG) reconstruction from this sample type. Third, the inherent susceptibility of Kraken2 k-mer-based classification to false-positive assignments, mitigated by stringent abundance cutoff and finally the relatively short sampling period (two seasons, five hospitals), which limits temporal and spatial generalizability. Future directions include expanding sampling to more hospitals and seasons; incorporating physicochemical parameters; applying higher sequencing depths; and developing risk assessment models and wastewater management strategies to mitigate ARG dissemination from Egyptian hospital wastewater.

## Conclusion

This study employed high-throughput metagenomic Nanopore sequencing to comprehensively characterize the bacterial community composition and resistome in hospital wastewater and tap water across five hospitals in Cairo, Egypt. Alpha and beta diversity analyses revealed significantly greater microbial diversity and distinct community composition in HWW compared to tap water, with *Acinetobacter* and *Propioniciclav* predominating in HWW and *Enterococcus*, *Escherichia*, and *Francisella* in tap water. The parallel application of three ARG databases (ResFinder, CARD, and NCBI AMRFinderPlus) demonstrated a high abundance and diversity of ARGs in HWW, particularly in Hospitals 1 and 5, while no ARGs were detected in tap water across all five hospitals. The most prevalent resistance mechanisms were associated with inhibition of protein synthesis and inhibition of metabolic pathways with aminoglycoside, macrolide, and tetracycline, resistance genes being the most predominant. The detection of 39 distinct plasmid replicons, predominantly of the Col family, highlights the significant potential for horizontal gene transfer and ARG dissemination from HWW into the broader environment. Furthermore, the detection of pathogenic genera in hospital tap water underscores critical public health implications and the urgent need for enhanced water quality surveillance, stringent antibiotic stewardship programs, and effective wastewater treatment strategies in Egyptian healthcare settings, in accordance with the One Health framework.

## Supplementary Information

Supplementary Information 1.

Supplementary Information 2.

Supplementary Information 3.

## Data Availability

All data generated or analyzed during this study are included in the published article and supplementary file. All row data of water sample sequences were deposited in the NCBI database under the accession number, PRJNA1266479 (https://www.ncbi.nlm.nih.gov/sra/PRJNA1266479).

## Abbreviations

AMR: Antimicrobial resistance
ARB: Antibiotic resistant bacteria
ARGs: Antibiotic resistance genes
HGT: Horizontal gene transfer
HWW: Hospital wastewater
MDR: Multiple drug resistance
MGEs: Mobile genetic elements
