# Supplementary figures and images for: Metagenomic profiling of microbial communities and the resistome within Egyptian hospital wastewater and tap water
Source: Sci Rep. 2026 Apr 30;16:13894. doi: 10.1038/s41598-026-49481-4 (PMC13133306; doi:10.1038/s41598-026-49481-4)

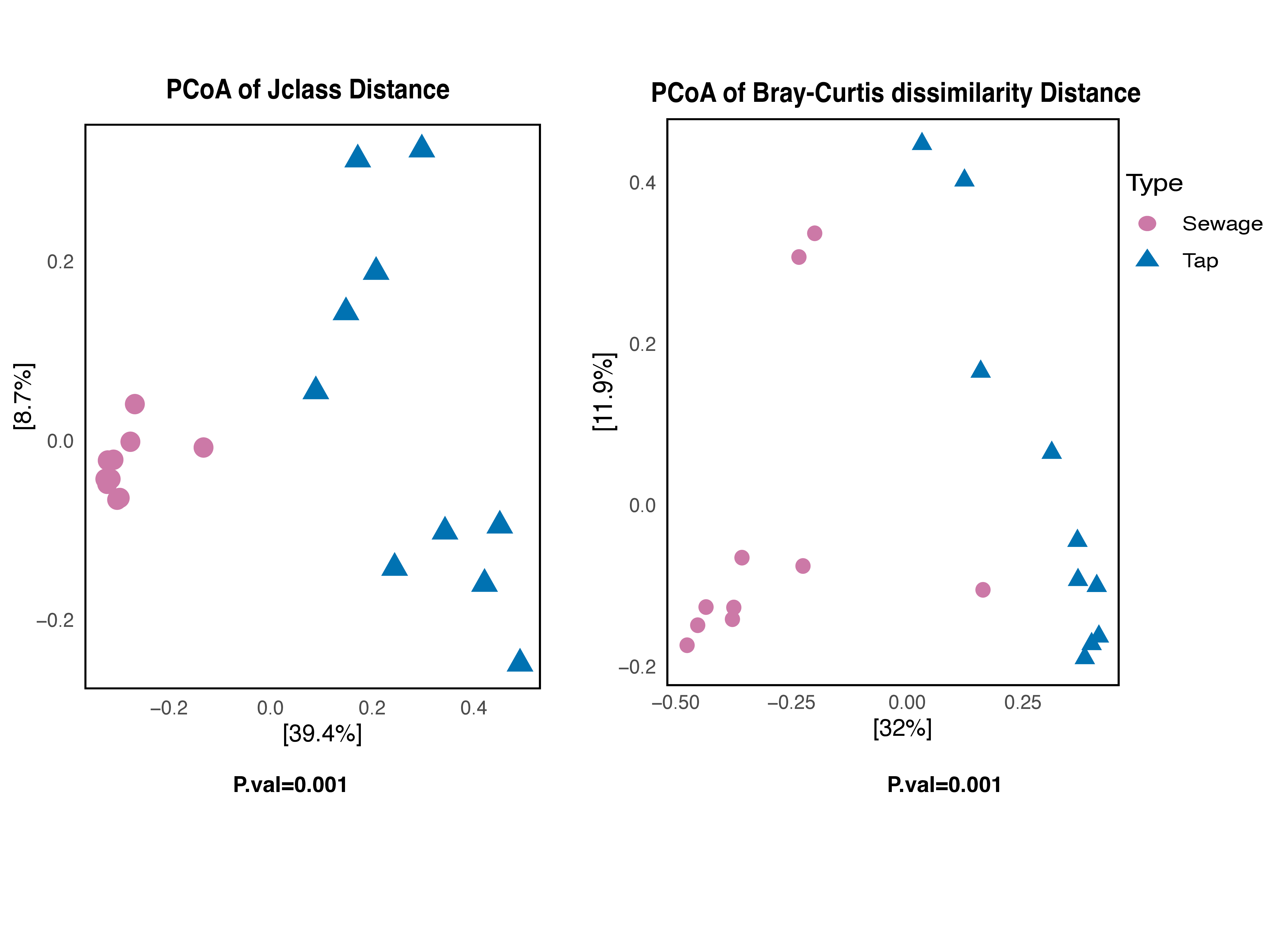

Supplement: Supplementary file 1 — Supplementary Information 1. [file 41598_2026_49481_MOESM1_ESM.tiff]

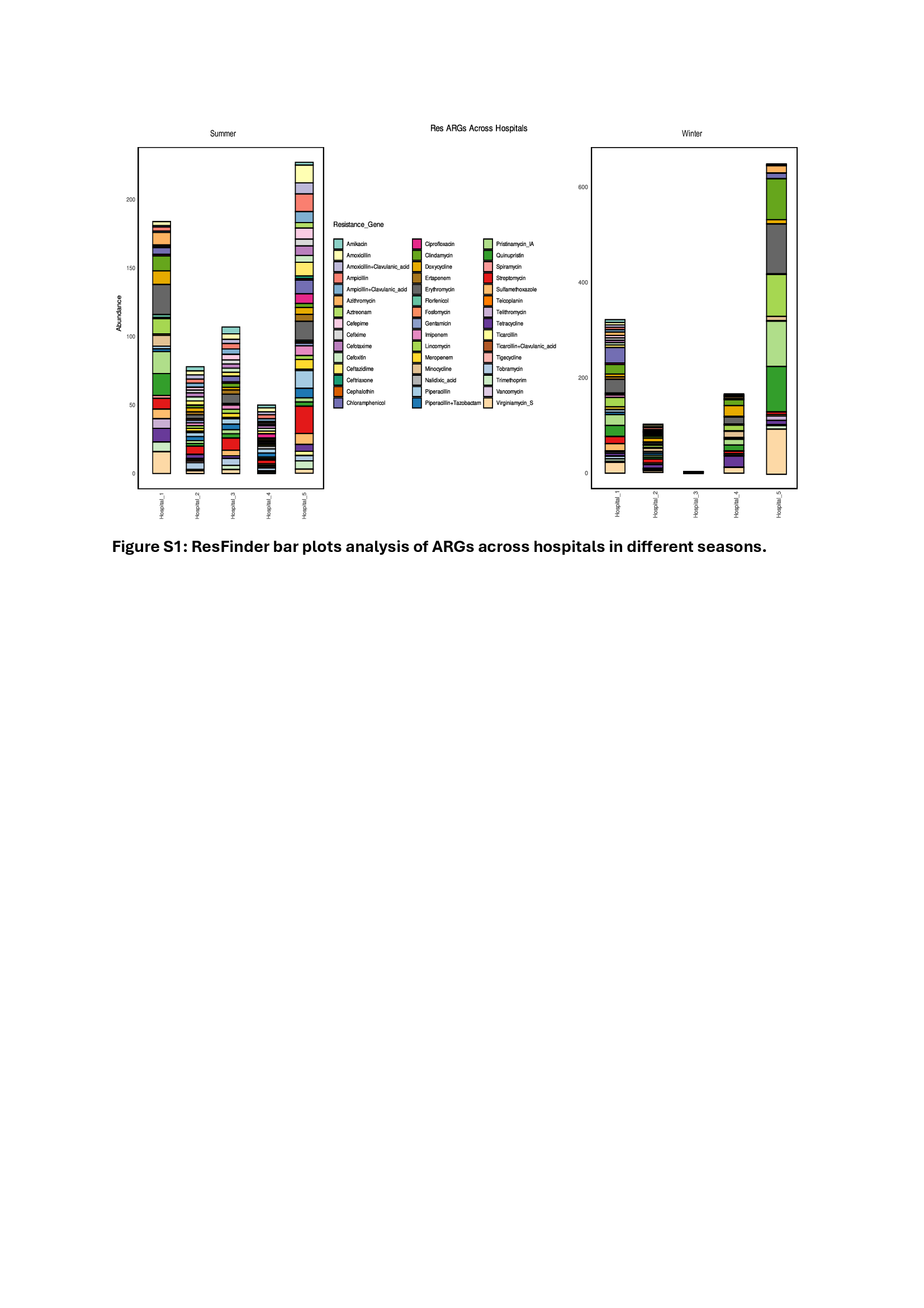

Supplement: Supplementary file 2 — Supplementary Information 2. [file 41598_2026_49481_MOESM2_ESM.tiff]
